# Alcohol Ingestion Impairs Maximal Post-Exercise Rates of Myofibrillar Protein Synthesis following a Single Bout of Concurrent Training

**DOI:** 10.1371/journal.pone.0088384

**Published:** 2014-02-12

**Authors:** Evelyn B. Parr, Donny M. Camera, José L. Areta, Louise M. Burke, Stuart M. Phillips, John A. Hawley, Vernon G. Coffey

**Affiliations:** 1 Exercise and Nutrition Research Group, School of Medical Sciences, RMIT University, Bundoora, Victoria, Australia; 2 Department of Sports Nutrition, Australian Institute of Sport, Canberra, ACT, Australia; 3 Exercise Metabolism Research Group, Department of Kinesiology, McMaster University, Hamilton, Ontario, Canada; 4 Exercise and Nutrition Research Group, School of Exercise Science, Australian Catholic University, Fitzroy, Victoria, Australia; 5 Research Institute for Sport and Exercise Sciences, Liverpool John Moores University, Liverpool, United Kingdom; 6 School of Exercise and Nutrition Sciences and Institute of Health and Biomedical Innovation, Queensland University of Technology, Kelvin Grove, Queensland, Australia; West Virginia University School of Medicine, United States of America

## Abstract

**Introduction:**

The culture in many team sports involves consumption of large amounts of alcohol after training/competition. The effect of such a practice on recovery processes underlying protein turnover in human skeletal muscle are unknown. We determined the effect of alcohol intake on rates of myofibrillar protein synthesis (MPS) following strenuous exercise with carbohydrate (CHO) or protein ingestion.

**Methods:**

In a randomized cross-over design, 8 physically active males completed three experimental trials comprising resistance exercise (8×5 reps leg extension, 80% 1 repetition maximum) followed by continuous (30 min, 63% peak power output (PPO)) and high intensity interval (10×30 s, 110% PPO) cycling. Immediately, and 4 h post-exercise, subjects consumed either 500 mL of whey protein (25 g; PRO), alcohol (1.5 g·kg body mass^−1^, 12±2 standard drinks) co-ingested with protein (ALC-PRO), or an energy-matched quantity of carbohydrate also with alcohol (25 g maltodextrin; ALC-CHO). Subjects also consumed a CHO meal (1.5 g CHO·kg body mass^−1^) 2 h post-exercise. Muscle biopsies were taken at rest, 2 and 8 h post-exercise.

**Results:**

Blood alcohol concentration was elevated above baseline with ALC-CHO and ALC-PRO throughout recovery (P<0.05). Phosphorylation of mTOR^Ser2448^ 2 h after exercise was higher with PRO compared to ALC-PRO and ALC-CHO (P<0.05), while p70S6K phosphorylation was higher 2 h post-exercise with ALC-PRO and PRO compared to ALC-CHO (P<0.05). Rates of MPS increased above rest for all conditions (∼29–109%, P<0.05). However, compared to PRO, there was a hierarchical reduction in MPS with ALC-PRO (24%, P<0.05) and with ALC-CHO (37%, P<0.05).

**Conclusion:**

We provide novel data demonstrating that alcohol consumption reduces rates of MPS following a bout of concurrent exercise, even when co-ingested with protein. We conclude that alcohol ingestion suppresses the anabolic response in skeletal muscle and may therefore impair recovery and adaptation to training and/or subsequent performance.

## Introduction

The focus of the early post-exercise period (i.e., 1–8 h) is to enhance physiological processes that are critical for reversing the exercise-induced disturbances to homeostasis and physiological function and for promoting adaptations to training [1]. Recommended nutritional strategies to maximize recovery in skeletal muscle include protein for enhancing rates of protein synthesis and carbohydrate for replenishing glycogen stores [2],[3]. Muscle contraction and the intake of leucine-rich protein sources activate independent but complimentary signaling responses that converge at the mechanistic target of rapamycin (mTOR) to stimulate protein translation enhancing rates of muscle protein synthesis [4]–[6]. The ingestion of ∼20–25 g of high quality protein soon after exercise [7], repeated every 4 h [8] has been shown to maximise the anabolic response in skeletal muscle.

The cultural environment surrounding some sports often involves the intake of large amounts of alcohol after training and competition, with athletes in several team sports being particularly at risk of “binge drinking” practices [9]–[11]. Indeed, a number of studies have reported that athletes are more likely than the general population to drink alcohol to excess, with a large proportion (∼50–65%) consuming intakes above the threshold classified as hazardous drinking [12], [13]. The outcomes of binge drinking after exercise are likely to include the direct effect of alcohol on physiological processes as well as the indirect effect on the athlete's recovery due to not eating or resting adequately as a result of intoxication. Although the concurrent consumption of carbohydrate can partially offset the deleterious effects of alcohol intake on post-exercise glycogen resynthesis [14], the effect of alcohol consumption on muscle protein synthesis is unknown.

Studies by Barnes and colleagues (2010, 2011) have investigated the effects of post-exercise alcohol consumption on human muscle function and performance [15], [16]. However, data on the effects of alcohol intake on skeletal muscle protein synthesis is limited to work in rodents. These studies show that both acute and chronic alcohol ingestion can have a detrimental effect on cell signaling and protein synthesis in skeletal muscle [17]–[21]. The aim of the current study was to determine the effect of alcohol intake on anabolic cell signaling and rates of myofibrillar protein synthesis (MPS) in humans during recovery from a bout of strenuous exercise approximating stresses an athlete may experience in training and performance for various team sports such as various football and rugby codes, and court sports. We hypothesized that compared to post-exercise protein intake, co-ingestion of alcohol would down-regulate translation initiation signaling and decrease rates of MPS.

## Methods

### Subjects

Eight healthy physically active male subjects (age 21.4±4.8 yr, body mass (BM) 79.3±11.9 kg, peak oxygen uptake (VO_2peak_) 48.1±4.8 mL·kg^−1^·min^−1^, leg extension one repetition maximum (1RM) 104±20 kg; values are mean ± SD) who had been participating in regular exercise (3 times wk^−1^ for >6 months) volunteered for this study. The experimental procedures and possible risks associated with the study were explained to each subject, who each gave written informed consent before participation.

### Ethics statement

All subjects were informed of the purpose of the study, the experimental procedures involved and all the potential risks involved before giving written consent. No minors were involved in this study as subjects were required to be 18 years of age at the time of participation due to the legal age for alcohol consumption in Australia. All subjects were deemed healthy based on their response to a routine medical screening questionnaire. The study was approved by the Human Research Ethics Committee of RMIT University (43/11) and was carried out according to the NHMRC National Statement on Ethical Conduct in Human Research (2007) and the Australian Code for the Responsible Conduct of Research (2007).

### Study Design

The study employed a randomized counter-balanced, cross-over design in which each subject completed bouts of consecutive resistance, continuous and intermittent high-intensity exercise with either post-exercise ingestion of alcohol-carbohydrate (ALC-CHO), alcohol-protein (ALC-PRO) or protein only (PRO) beverages on three separate occasions. Each experimental trial was separated by a two week recovery period, during which time subjects maintained their habitual physical activity pattern. Given the data showing little/no effect of carbohydrate ingestion on myofibrillar protein synthesis, the ALC-CHO treatment was used as an iso-energetic control. The decision not to use parallel groups was based on a within subject crossover design adding strength to the interpretation and conclusions of the study but limited the total number of treatments such that an exercise only trial was not undertaken. Finally, we based our exercise protocol incorporating the different metabolic stresses approximating those experienced in team sports due to published reports of the increased incidence of excessive alcohol consumption following performance in team/group sport [13].

### Preliminary Testing

#### VO_2_peak

VO_2peak_ and peak power output (PPO) were determined during an incremental test to volitional fatigue on a Lode cycle ergometer (Groningen, The Netherlands). The protocol has been described in detail previously [22].

#### Maximal strength

Quadriceps strength was determined on a plate-loaded leg extension machine until the 1RM load was established. Repetitions were separated by a 3-min recovery and were used to establish the maximum load/weight that could be moved through the full range of motion once, but not a second time.

### Diet/exercise control

For the 48 h prior to an experimental trial subjects were instructed to refrain from strenuous exercise/training. Subjects were provided pre-packaged food and drinks (∼6000 kJ; 3.1 g CHO·kg^−1^ BM, 0.5 g fat·kg^−1^ BM, 0.4 g protein·kg^−1^ BM) to be consumed for the last meal prior to an experiment. A food diary to record dietary intake was used to ensure adherence to the final meal and overall daily intake for the 24 h prior to an experiment day.

### Experimental Procedure

**Figure 1:**
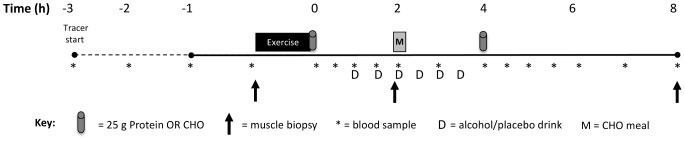
Schematic representation of the experimental trial. Subjects reported to the laboratory after an overnight fast where a constant infusion of L-[ring-^13^C_6_] phenylalanine was commenced (3 h in first trial; 1 h in trial 2/3), and subjects completed the concurrent exercise (8×5 repetitions at 80% one repetition maximum (1RM), 5 min rest, 30 min cycling at ∼63% peak power output (PPO), 2 min rest, 10×30 s high intensity intervals at ∼110% PPO). Immediately after exercise completion, and 4 h later, subjects consumed 500-mL of protein (25 g whey) or carbohydrate (25 g maltodextrin).

The study employed a randomized cross-over design in which each subject completed three experimental trials. Each trial was separated by 14 d, during which subjects maintained their habitual level of physical activity and their normal diet. The three trials compared post-exercise protein synthesis with three different treatments: a post-exercise feeding regimen providing protein intake for optimal muscle protein synthesis [8] (2 feedings of 25 g high quality protein at 0 and 4 h of recovery: PRO), a trial in which the subjects consumed 1.5 g·kg^−1^ BM ethanol plus an energy match for recommended protein feedings in the form of carbohydrate (ALC-CHO), and ALC-PRO in which the same amount of alcohol was consumed in addition to protein intake in PRO also ingested at 0 and 4 h post-exercise (see Figure 1). All trials involved a further standardised carbohydrate-rich meal (1.5 g CHO·kg^−1^ BM) at 2 h post-exercise as post-event fuelling/eating.

On the morning of an experimental trial, subjects reported to the laboratory after a ∼10-h overnight fast. After resting in a supine position for ∼15 min, catheters were inserted into the antecubital vein of each arm and a baseline blood sample (∼4 mL) was taken from one arm. A primed constant intravenous infusion (prime: 2 µmol·kg^−1^; infusion 0.05 µmol·kg^−1^ min^−1^) of L-[*ring*-^13^C_6_] phenylalanine (Cambridge Isotopes Laboratories, Woburn, MA, USA) was then administered to the contralateral arm for the duration of the experiment. Under local anaesthesia (1% Xylocaine) a resting biopsy from the *vastus lateralis* of one leg was obtained 3 h after commencement of the tracer infusion using a 5-mm Bergstrom needle modified with suction, during the first trial only. This procedure was undertaken once during subjects first experimental trial to obtain resting fractional synthetic rates using the previously validated single biopsy method [23]. During subsequent trials tracer infusion commenced 1 h prior to the exercise protocol. The exercise bout incorporated the concurrent stimuli of resistance, continuous and intermittent high-intensity exercise to represent the key features of team sport activities. The specific protocol involved a standardized warm-up (5 repetitions at 50% and 5 repetitions at 60% 1RM) on a leg extension machine before the resistance exercise protocol was commenced. Resistance exercise consisted of eight sets of five repetitions at ∼80% of 1RM. Each set was separated by a 3-min recovery period during which the subject remained seated. After completion of the final set, subjects rested for 5 min before commencing 30 min of continuous cycling at ∼63% PPO (∼70% VO_2peak_). Upon completion, subjects rested on the bike for 2 min before undertaking 10×30 s high intensity intervals at ∼110% of PPO, with 30 s active recovery (∼50% PPO) between each work bout.

Immediately following exercise and after 4 h recovery, subjects ingested a 500 mL solution of either protein (PRO, 25 g whey protein powder; ISO8, Musashi, Melbourne, VIC Australia) or an energy-match in the form of CHO (25 g maltodextrin, International Health Investments, Helensvale, QLD Australia). Furthermore, a CHO-based meal (1.5 g·kg^−1^ BM) was consumed ∼2 h post-exercise, immediately after the muscle biopsy, according to recommendations for post-exercise glycogen recovery [24]. Protein beverages included L-(*ring*-^13^C_6_] phenylalanine at 4% to prevent marked disturbance in isotopic enrichment and to maintain steady state enrichment. Blood (∼4 mL) was collected immediately post-exercise and at regular intervals (30–60 min) throughout an 8 h recovery period, with additional muscle biopsies from separate incisions taken at 2 and 8 h post-exercise. Samples were stored at −80°C until analysis. The 8 h time frame represents an important phase of post-exercise recovery [1] as well as the period during which blood alcohol concentrations are likely to be elevated by a post-event drinking binge [14]. The alcohol dose in the present study represented the mean intake of alcohol reported by team athletes during a drinking binge [9], [10] and an amount previously investigated in relation to post-exercise refuelling [14]. The alcohol ingestion protocol (1.5 g·kg^−1^ BM; 12±2 standard drinks) began 1 h post-exercise and was consumed in 6 equal volumes of 1 part vodka (∼60 mL) to four parts orange juice (∼240 mL, 1.8 g CHO·kg^−1^ BM) during a 3 h period. For the PRO condition, orange juice was consumed with a matched volume of water in place of the alcohol. Subjects ingested the beverages within 5 min every 30 min.

### Analytical Procedures

#### Blood glucose and plasma ethanol concentrations

Whole blood samples (∼25 µL) were immediately analysed for glucose concentrations using an automated analyser (YSI 2300, Yellow Springs, OH, USA). Blood samples were then centrifuged at 3,000 *g* at 4°C for 10 min, with aliquots of plasma frozen and stored at −80°C. On a separate occasion, plasma samples (∼25 µL) were thawed and analysed for ethanol concentration using an automated analyser (YSI 2900, Yellow Springs, OH, USA).

#### Plasma amino acids and enrichment

Plasma amino acid concentrations and enrichments were analyzed by gas chromatography-isotope ratio mass spectrometry (MAT252; Finnigan, Breman, Germany) using EZ:faast kit (Phenomenex, CA, USA).

#### Rates of Myofibrillar Protein Synthesis

A single pre-infusion plasma sample, extracted by acetonitrile, was utilized as the baseline enrichment in tracer naïve subjects [23]. For the one non-tracer naïve subject a pre-infusion muscle biopsy was used for baseline enrichment. Muscle tissue was processed as previously described [7].

#### Calculations

The fractional synthetic rate (FSR) of myofibrillar protein synthesis was calculated using the standard precursor–product method: 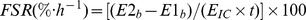



Where E2_b_ - E1_b_ represents the change in the bound protein enrichment between two biopsy samples; E_IC_ is the average enrichment of intracellular phenylalanine between the two biopsy samples; and *t* is the time between two sequential biopsies. The inclusion of ‘tracer-naive’ subjects permitted use of the pre-infusion blood sample (i.e. single biopsy method) as the baseline enrichment (E1_b_) for the calculation of resting MPS.

#### Western Blots

Intracellular signaling proteins were extracted, isolated and quantified as previously described [25]. The amount of protein loaded in each well was 50 µg. Polyclonal anti-phospho mechanistic target of rapamycin (mTOR) Ser2448 (no. 2971), elongation factor 2 (eEF2) Thr56 (no. 2331), 4E-BP1 Thr37/46 (no. 2855), monoclonal anti- 5′ adenosine monophosphate-activated protein kinase (AMPK) α Thr172 (no. 2535) and p70S6K Thr389 (no. 9234) were from Cell Signalling Technology (Danvers, USA). Data represent the volume and intensity quantified via densitometry and phosphorylation data and are expressed relative to α-tubulin reference protein expression at the equivalent time point on the same membrane (no. 3873, Cell Signalling Technology, Danvers, USA) in arbitrary units. All samples for each subject were run on the same gel.

#### Real Time PCR

Skeletal muscle (∼20 mg) tissue RNA extraction, reverse transcription and real-time polymerase chain reaction (RT-PCR) was performed as previously described [25]. TaqMan-FAM labeled primer/probes (Applied Biosystems, Carlsbad, CA, USA) for muscle ring finger 1 (MuRF-1) (Cat No. Hs00261590) and Atrogin (Cat No. Hs01041408) were used in a final reaction volume of 20 µL. Glyceraldehyde- 3-phosphate dehydrogenase (GAPDH, HS9999- 9905_m1) was used as the housekeeping gene. The relative amounts of mRNAs were calculated using the relative quantification (ΔΔCT) method [26].

### Statistical Analysis

Blood, cell signaling and mRNA data were analyzed by two-way ANOVA (two factor: time × treatment) with repeated measures and myofibrillar protein synthesis was analyzed by one-way ANOVA with repeated measures. All data underwent Student-Newman-Keuls post hoc analysis when P<0.05 (SigmaPlot for Windows; Version 12.5). All data are expressed as mean ± SD and the level of statistical significance was set at P<0.05.

## Results

### Blood Alcohol and Glucose Concentration

**Figure 2:**
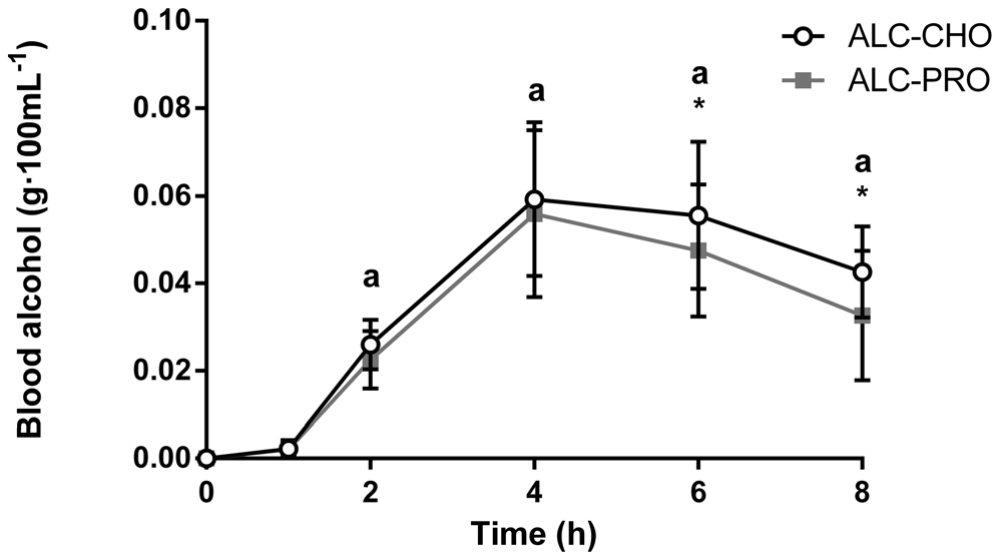
Blood alcohol levels after alcohol intake during recovery following a single bout of concurrent training. Data were analysed using a 2-way ANOVA with repeated measures and Student-Newman-Keuls post hoc analysis. Values are mean ± SD. Significant effect of treatment (P = 0.02), time (P<0.01) with no interaction (P = 0.20). Significantly different (P<0.05) vs. (a) rest, and (*) between treatments (ALC-CHO vs. ALC-PRO).

**Figure 3:**
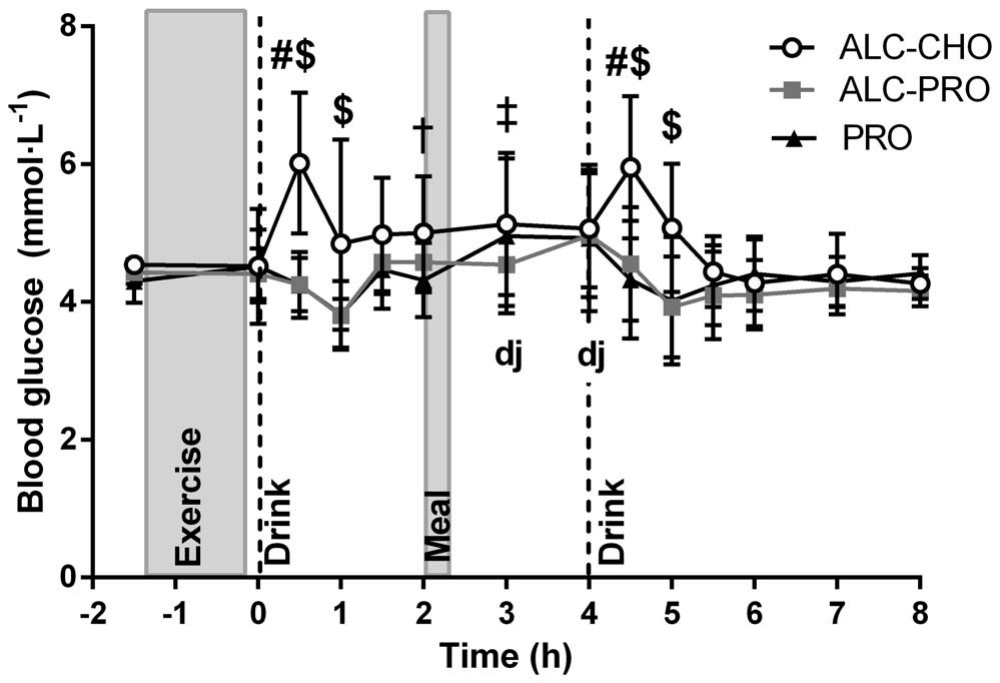
Blood glucose concentrations before and duringrecovery following a single bout of concurrent training. Drink  =  25 g of whey protein (PRO and ALC-PRO) or 25 g maltodextrin (ALC-CHO); Meal  =  1.5 g·kg^−1^ BM. Data were analysed using a 2-way ANOVA with repeated measures and Student-Newman-Keuls post hoc analysis. Values are mean ± SD. Significant effect of treatment, time and interaction (all P<0.01). Significantly different (P<0.05) (d) from 1 h within treatment, (j) from 5 h within treatment, ($) between treatments (ALC-CHO vs. ALC-PRO, PRO). (†) between treatments (ALC-CHO vs. PRO), (‡) between treatments (ALC-CHO vs. ALC-PRO).

There were main effects for time and treatment for blood alcohol concentration (P<0.05; Figure 2). Blood alcohol concentration peaked 4 h post-exercise (ALC-CHO 0.059±0.017 g·100 mL^−1^; ALC-PRO 0.056±0.019 g·100 mL^−1^) and remained elevated above rest throughout the 8 h recovery period (ALC-CHO: 0.023–0.059 g·100 mL^−1^; ALC-PRO: 0.029–0.056 g·100 mL^−1^; P<0.05). Blood alcohol concentration was higher (P<0.05) with ALC-CHO compared with ALC-PRO at 6 h (ALC-CHO: 0.055 g·100 mL^−1^; ALC-PRO: 0.047 g·100 mL^−1^) and 8 h (ALC-CHO: 0.043 g·100 mL^−1^; ALC-PRO: 0.033 g·100 mL^−1^) post-exercise. Blood glucose concentration increased above all time-points at 0.5 h (∼17–41%) and 4.5 h (∼16–40%) in the ALC-CHO treatment (P<0.05; Figure 3) but was not different from resting in ALC-PRO and PRO treatments. The blood glucose concentration measured in the ALC-CHO treatment was also different from ALC-PRO (∼27–41%) and PRO (∼26–42%) at 0.5, 1, 4.5 and 5 h post-exercise (P<0.05).

### Plasma Amino Acids Concentration

**Figure 4:**
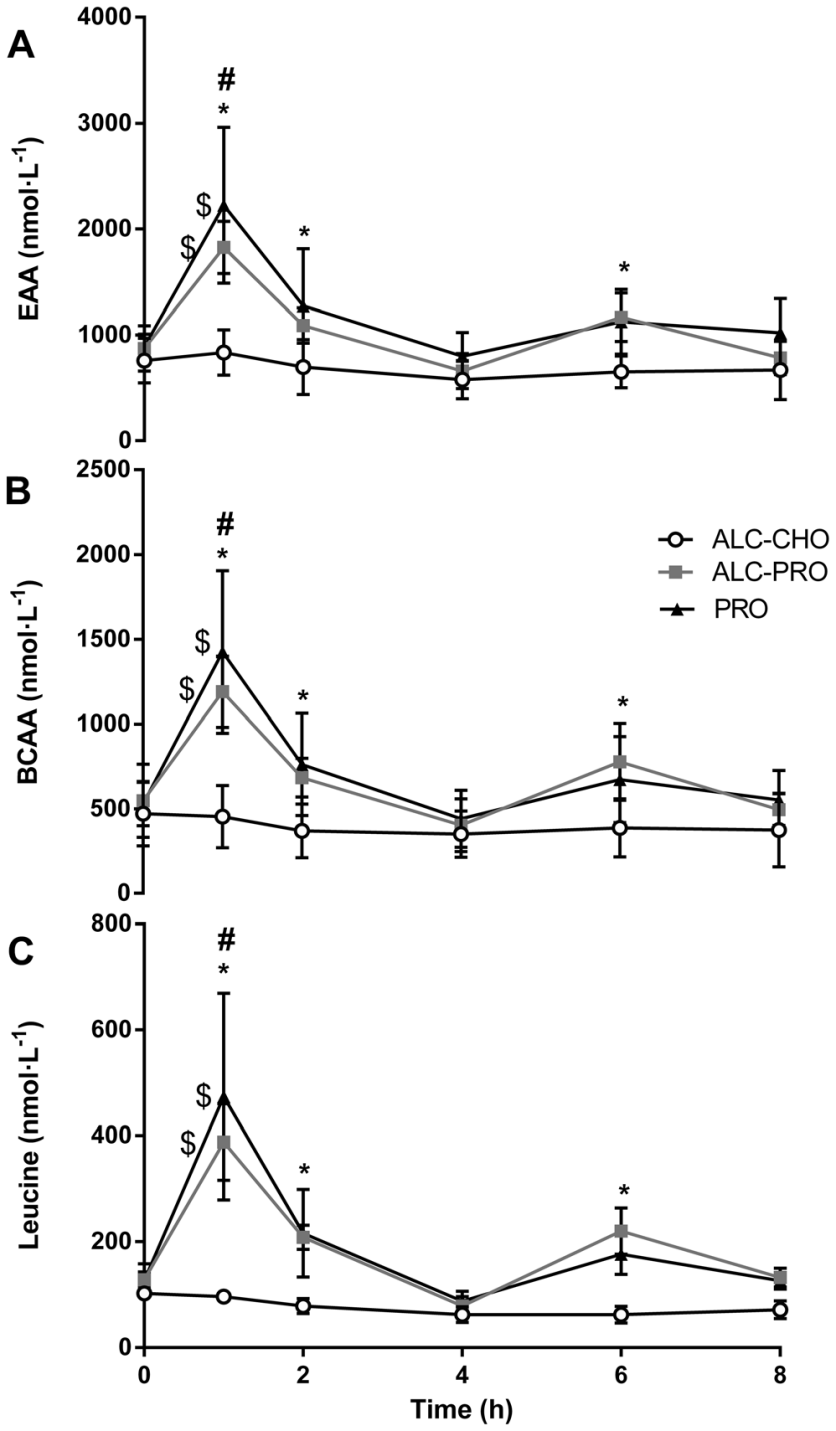
Plasma EAA (A), BCAA (B), leucine (C) concentration following a single bout of concurrent training. EAA – essential amino acids; BCAA – branched-chain amino acids. Data were analysed using a 2-way ANOVA with repeated measures and Student-Newman-Keuls post hoc analysis. Values are mean ± SD. Significant effect of treatment, time and interaction (all P<0.01) for (A), (B), and (C). Significantly different (P<0.05) vs. (#) all timepoints for ALC-CHO and ALC-PRO treatments, (*) vs. rest within treatments, and ($) compared to ALC-CHO.

There were main effects for time and treatment for plasma EAA, BCAA and leucine concentrations (P<0.05; Figure 4). Protein intake increased AA concentration at 1 h post-exercise: AA concentrations for ALC-PRO (EAA ∼109%, BCAA ∼118%, leucine ∼203%) and PRO (EAA ∼151%; BCAA ∼170%; leucine ∼274%) treatments were different to all other time-points within treatments (P<0.05). Post-exercise concentrations of EAA and leucine with PRO were elevated above resting values at 1 h (∼39%), 2 h (∼98%) and 6 h (∼61%) time-points, respectively (P<0.05). Leucine concentration remained above resting values 2 h (∼90%) and 6 h (∼102%) post-exercise, and EAA and BCAA were also higher than rest after 6 h recovery (EAA ∼77%; BCAA ∼38%) in the ALC-PRO treatment (P<0.05). Compared to ALC-CHO treatments, AA concentration were higher for ALC-PRO and PRO at 1 h (ALC-PRO: ∼115–305%, PRO: ∼163–394%), 2 h (∼56–168%, ∼83–179%) and 6 h (∼81–253%, ∼75–181%) post-exercise time-points (P<0.05). There were no changes in AA concentration in the ALC-CHO treatment.

### Intracellular and Plasma Tracer Enrichments

Phenylalanine enrichments showed a stable precursor pool throughout the infusion period in all groups (Figure S1). Linear regression analysis indicated that the intracellular (mean r^2^ = 0.08) and plasma (mean r^2^ = 0.03) enrichments in ALC-CHO, ALC-PRO and PRO treatments demonstrated isotopic plateau.

### Cell Signaling

#### mTOR-p70S6K

**Figure 5:**
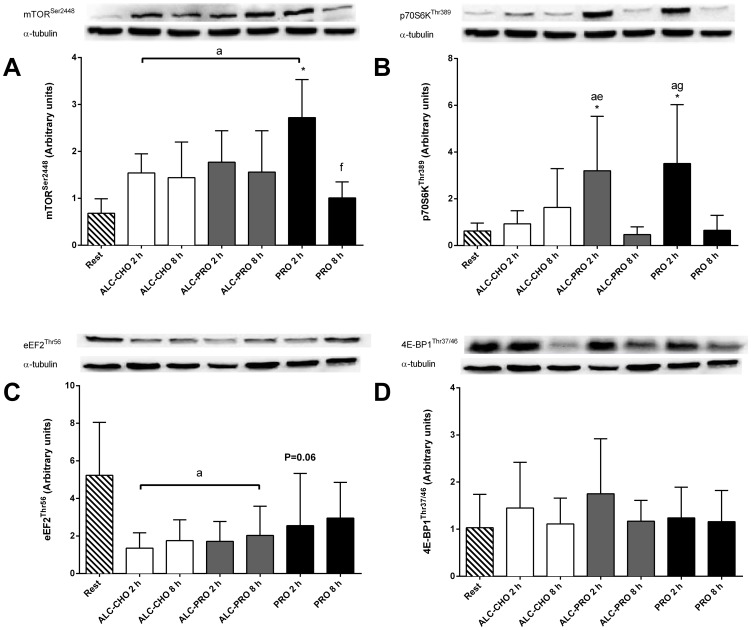
mTOR^Ser2448^ (A), p70S6K^Thr389^ (B), eEF2^Thr56^ (C), 4E-BP1^Thr37/46^ (D) phosphorylation at rest and following a single bout of concurrent training. Images are representative blots for each protein from the same subject and values are expressed relative to α-tubulin and presented in arbitary units. Data were analysed using a 2-way ANOVA with repeated measures with Student-Newman-Keuls post hoc analysis. Values are mean ± SD. Significant effect of time (P<0.01) and interaction (P = 0.02) but not treatment (P = 0.22) for (A); time (P<0.01) and interaction (P = 0.02) but not treatment (P = 0.46) for (B); time (P<0.01) but not treatment (P = 0.14) or interaction (P = 0.56) for (C); no treatment (P = 0.86), time (P = 0.24), or interaction (P = 0.77) effects for (D). Significantly different (P<0.05) vs. (a) rest, (e) ACL-PRO 8 h, (f) PRO 2 h, (g) PRO 8 h, and (*) 2 h between treatments.

There were main effects for time and treatment for mTOR^Ser2448^ phosphorylation (P<0.05, Figure 5A). mTOR phosphorylation increased above rest at 2 h (P<0.05) for all treatments (ALC-CHO: ∼125%, ALC-PRO: ∼157%, PRO: ∼297%) and at 8 h (P<0.05) for ALC-CHO (∼111%) and ALC-PRO (∼127%). mTOR phosphorylation with PRO was higher (P<0.05) than ALC-CHO (∼76%) and ALC-PRO (∼54%) at 2 and 8 h post-exercise, and PRO at 8 h post-exercise (∼168%).

There were main effects for time and treatment for p70S6K^Thr389^ phosphorylation (P<0.05, Figure 5B). p70S6K phosphorylation was greater at 2 h (P<0.05) compared to rest and 8 h post–exercise in ALC-PRO (∼418–585%) and PRO only (∼438–468%). p70S6K phosphorylation was also higher at 2 h post-exercise in PRO (∼276%) and ALC-PRO (∼242%) treatments compared to ALC-CHO treatment (P<0.05).

#### eEF2-4E-BP1-AMPK

There were decreases in eEF2 phosphorylation (Figure 5C) below rest at 2 h and 8 h post-exercise in ALC-CHO (∼66–74%; P<0.05) and ALC-PRO (∼61–67%; P<0.05). No changes in 4E-BP1^Thr37/46^ (Figure 5D) or AMPK^Thr172^ phosphorylation [data not shown] were observed across treatments or times.

### Atrogene mRNA expression

**Figure 6:**
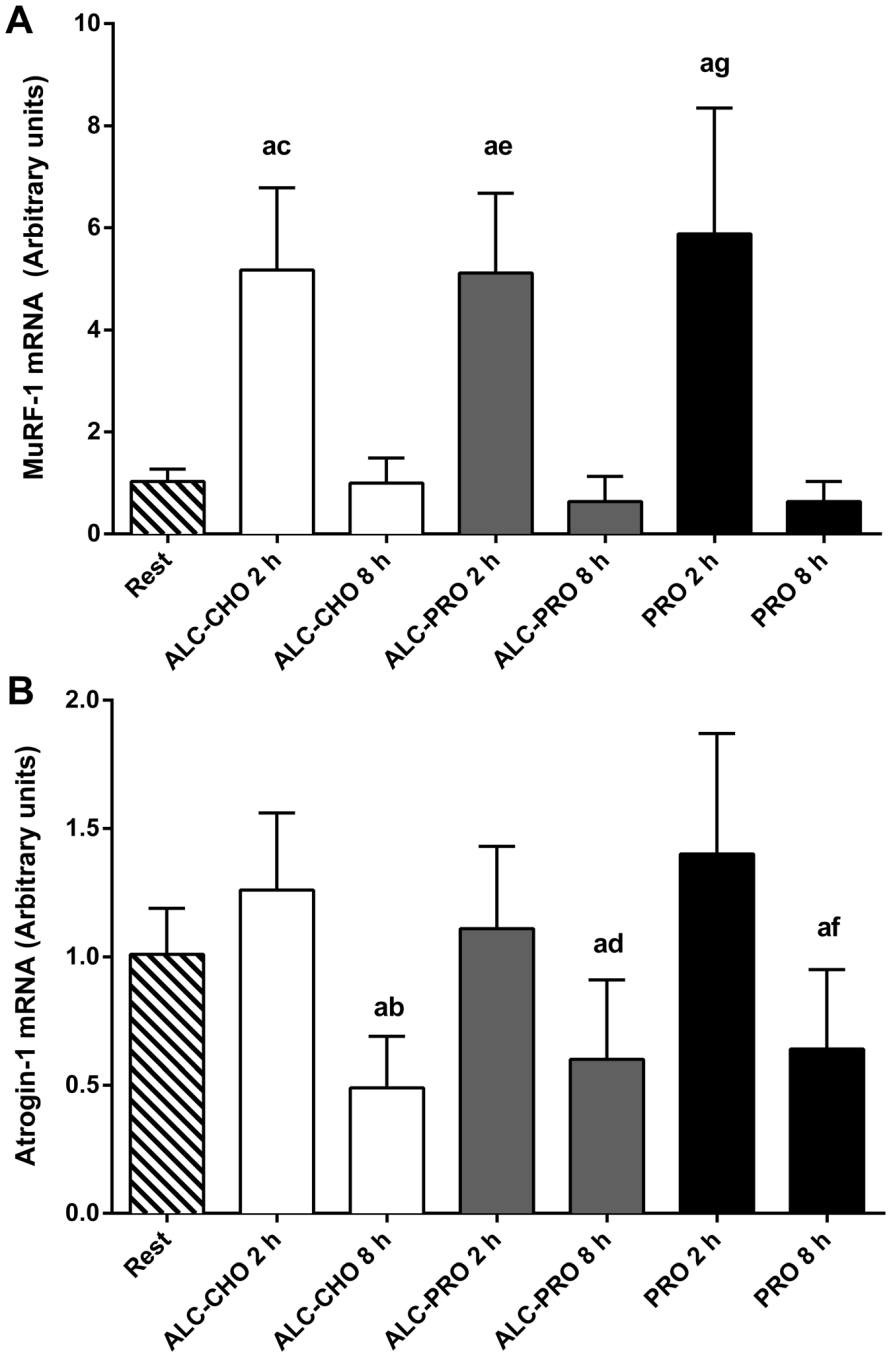
MuRF-1 (A), Atrogin-1 (B) mRNA abundance at rest and following a single bout of concurrent training. Values are expressed relative to GAPDH and presented in arbitrary units (mean ± SD, n = 7). Data were analysed using a 2-way repeated measures ANOVA with Student-Newman-Keuls post hoc analysis. Significantly different (P<0.05) vs. (a) rest, (c,e,g) 8 h within treatments, and (b,d,f) 2 h within treatments.

There were increases above rest in MuRF-1 mRNA (Figure 6A, P<0.05) at 2 h for all treatments (ALC-CHO: ∼404%; ALC-PRO: ∼399%; PRO: ∼474%). However, there were no differences between treatments, and MuRF1 mRNA returned to resting levels under all conditions after 8 h (P<0.05). There was a main effect for time for atrogin-1 abundance (P<0.05, Figure 6B). Atrogin-1 mRNA expression at 8 h decreased below rest (∼37–52%; P<0.05) and 2 h (∼46–61%; P<0.05) with all treatments.

### Rates of muscle protein synthesis

**Figure 7:**
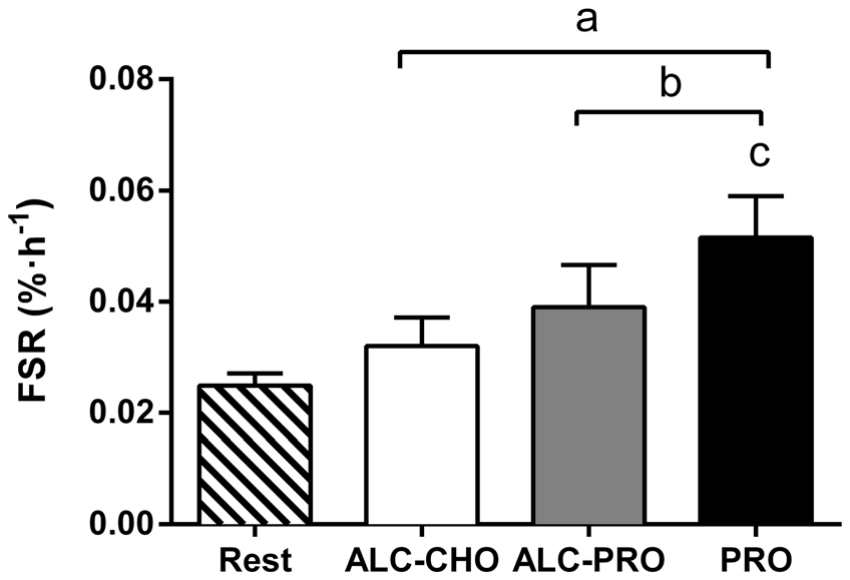
Myofibrillar fractional synthetic rate (FSR) throughout 2–8 h recovery following a single bout of concurrent training. Data were analysed using a 1-way repeated measures ANOVA with Student-Newman-Keuls *post hoc* analysis. Values are mean ± SD expressed as % h^−1^, n = 8. Significantly different (P<0.05) vs. (a) rest, (b) ALC-CHO, (c) ALC-PRO.

Rates of myofibrillar FSR were increased above rest (0.025±0.002%·h^−1^) with ALC-CHO (0.032±0.005%·h^−1^, ∼29%), ALC-PRO (0.039±0.008% h^−1^, ∼57%) and PRO (0.052±0.008%·h^−1^, ∼109%) treatments throughout 2–8 h of recovery (P<0.05; Figure 7). However, compared to PRO alone, there was a hierarchical reduction in myofibrillar FSR with ALC-PRO (24%, P<0.05) and ALC-CHO (37%, P<0.05). ALC-CHO resulted in a lower FSR compared to ALC-PRO (∼18%, P<0.05).

## Discussion

The first novel finding of this study was that mTOR signaling and rates of myofibrillar protein synthesis (MPS) following concurrent resistance, continuous and intermittent high-intensity exercise, designed to mimic the metabolic profile of many team sports, were impaired during the early (8 h) recovery phase by the ingestion of large amounts (1.5 g•kg^−1^ BM) of alcohol. These outcomes were most evident (37% reduction in rates of MPS) when alcohol was consumed in the absence of post-exercise protein intake, as is likely to occur when intoxication reduces the athlete's compliance to sound recovery practices. However, a second finding was that even when protein was consumed in amounts shown to be optimally effective to stimulate MPS [8] during post-exercise recovery, the intake of alcohol reduced MPS by ∼24%, representing only a partial ‘rescue’ of the anabolic response compared with protein alone.

The alcohol consumption protocol used in the current study, representing the mean intake of alcohol that has been self-reported in several studies of binge drinking practices of team athletes [9], [10], elicited blood alcohol concentrations that exceeded the 0.05 g·100 mL^−1^ legal limit for driving in Australia (Figure 1). Although peak post-exercise blood alcohol values were lower than we have previously reported [14], such differences can, in part, be explained by different alcohol ingestion protocols and feeding regimens. The subtle differences in blood alcohol concentration were likely a result of the different macronutrient composition consumed and the aminoacidemia in PRO and ALC-PRO was similar and significantly different to that seen with the carbohydrate treatment (Figure 4).

Despite alcohol having little effect on blood amino acid profiles, myofibrillar FSR was significantly different between treatments (Figure 7). The maximal FSR was measured when protein was the only nutrient ingested, and is similar to other studies incorporating resistance-type exercise with protein feeding [25], [27]. However, this study is the first to have measured FSR after consecutive bouts of resistance, continuous and high-intensity exercise when alcohol was consumed during recovery. While several studies examining the effects of alcohol intake have been undertaken in rodents, the relative quantity of alcohol administered in these investigations is several fold higher than in the current human study [18], [21], [28]–[30]. Furthermore, there are differences in techniques used to measure rates of protein synthesis in animals versus humans. Notwithstanding these differences, Lang et al. [28] reported a 25% decrease in rates of muscle protein synthesis with alcohol administration in rodents, a value in close agreement with the current study. Our results show alcohol ingestion in humans suppresses the elevated rates of protein synthesis in skeletal muscle induced by exercise and protein ingestion.

The mechanistic target of rapamycin complex 1 (mTORC1) is a central node for integrating nutrient (i.e. amino acid) and exercise/contraction signal transduction [31], [32]. Post-exercise phosphorylation of mTOR^Ser2448^ was attenuated when alcohol was co-ingested with either carbohydrate or protein compared to protein ingestion alone. Interestingly, there was discordance in phosphorylation responses between mTOR and its downstream signaling targets (p70S6K and 4E-BP1). The mechanism through which alcohol may attenuate mTOR complex 1 activity is still poorly defined. Recent evidence has implicated several upstream regulatory mechanisms of mTOR signaling including the Rag family of GTPases [33], [34], phosphatidic acid [35] and the DNA damage response 2 (REDD2) protein [36]. The inhibitory effects of alcohol on mTOR phosphorylation in skeletal muscle have been attributed to increases in the mRNA/protein content of the negative mTOR regulator REDD1 with acute intoxication and that alcohol may also generate greater association of mTOR with raptor to down regulate mRNA translation [20], [37]. Thus it is plausible that several mechanisms may act synergistically upstream of mTOR in response to alcohol ingestion to modulate mTOR activity. Nevertheless, our findings indicate that the observed alcohol-induced attenuation of MPS was likely mediated, at least in part, by effects on mTORC1-mediated signaling.

p70S6K enhances translation of mRNAs encoding ribosomal proteins and elongation factors [38] and has been proposed as a ‘surrogate’ marker associated with rates of muscle protein synthesis [39]–[42]. Lang and co-workers have previously shown reduced p70S6K signaling following alcohol ingestion in rat skeletal muscle [18], [29]. We present new information in human skeletal muscle to demonstrate the exercise and nutrient-induced increase in p70S6K phosphorylation is significantly reduced with alcohol ingestion in the absence of the co-ingestion of protein. The discordant mTOR-p70S6K phosphorylation with protein only and protein feedings with alcohol is not unprecedented given we [8] and others [43] have shown that mTOR-S6K phosphorylation often parallels changes in MPS but does not always reflect either the magnitude or duration of the increased MPS signal in humans. An alternate mechanism through which alcohol may limit rates of protein synthesis is endoplasmic reticulum stress and the resultant unfolded protein response. Alcohol consumption generates oxidative stress and inflammation and the potential to disrupt endoplasmic reticulum homeostasis; a consequence of this response is to limit the rate of protein synthesis [44], [45]. The lack of change in 4E-BP1^Thr37/46^ phosphorylation following exercise and between treatments contrasts previous findings in rodents [18], [20], [28]. However, these differences may, in part, be explained by the 2.3 fold greater relative alcohol administration in rodents versus humans. Finally, it must be acknowledged that our data are potentially limited by providing only a single ‘snapshot’ during recovery and the possibility exists that our muscle biopsy time-points failed to coincide with peak phosphorylation responses of signal transduction. To the best of our knowledge, this is the first study to investigate the effect of alcohol ingestion following concurrent resistance, continuous and intermittent high-intensity exercise in human skeletal and further studies are needed to better understand the precise mechanisms through which alcohol attenuates human skeletal muscle protein synthesis.

In contrast with the changes in cell signaling, muscle mRNA responses of selected genes associated with muscle proteolysis and catabolism were largely unchanged between treatments. MuRF-1 mRNA expression was elevated 2 h following exercise but had returned to basal levels, by 8 h in all treatments. Whereas, atrogin-1 mRNA expression did not change 2 h following exercise and was significantly lower than rest and 2 h post-exercise at 8 h post-exercise in all treatments. These results contrast findings by Vary and colleagues [30] who found alcohol ingestion to increase MuRF-1 and Atrogin-1 mRNA abundance in rat skeletal muscle. Our data shows protein co-ingested with alcohol following exercise induces comparable increases in atrogene mRNA expression compared to protein ingestion alone in human skeletal muscle. These increases are in agreement with previous findings demonstrating increased atrogene mRNA expression following resistance exercise [46], [47]. Although we did not determine rates of muscle protein breakdown, this process is up-regulated in mixed muscle for up to 24 h after resistance exercise in the fasted state [48]. As muscle damaging exercise has previously been reported to decrease GLUT4 translocation and subsequent rates of muscle glycogen resynthesis [49], the possibility that it also may impart a negative effect on protein transporters and rates of protein synthesis cannot be discounted. However, the atrogene results of the current study indicate alcohol ingestion does not exert any additional effects on ubiquitin ligase expression after exercise in human skeletal muscle. Future studies investigating the time course of atrogene expression and direct measures of skeletal muscle proteasome activity and/or protein breakdown following alcohol ingestion in humans are warranted.

In conclusion, the current data provide the novel observation that alcohol impairs the response of MPS in exercise recovery in human skeletal muscle despite optimal nutrient provision. The quantity of alcohol consumed in the current study was based on amounts reported during binge drinking by athletes. However, published reports suggest intakes of some individuals can be significantly greater [9], [50], which is of concern for many reasons related to health and safety [13]. Regrettably, there has been difficulty in finding an educational message with alcohol consumption related to sports performance that has resonance with athletes. Given the need to promote protein synthesis that underpins adaptation, repair and regeneration of skeletal muscle the results of the current study provide clear evidence of impaired recovery when alcohol is consumed after concurrent (resistance, continuous and intermittent high-intensity) exercise even in the presence of optimal nutritional conditions. We propose our data is of paramount interest to athletes and coaches. Our findings provide an evidence-base for a message of moderation in alcohol intake to promote recovery after exercise with the potential to alter current sports culture and athlete practices.

## Supporting Information

Figure S1 **Tracer enrichment of the muscle intra-cellular protein pool (A) and blood plasma (B) following a single bout of concurrent training.**
(TIF)
