# Supplementary figures and images for: Alcohol Ingestion Impairs Maximal Post-Exercise Rates of Myofibrillar Protein Synthesis following a Single Bout of Concurrent Training
Source: PLoS One. 2014 Feb 12;9(2):e88384. doi: 10.1371/journal.pone.0088384 (PMC3922864; doi:10.1371/journal.pone.0088384)

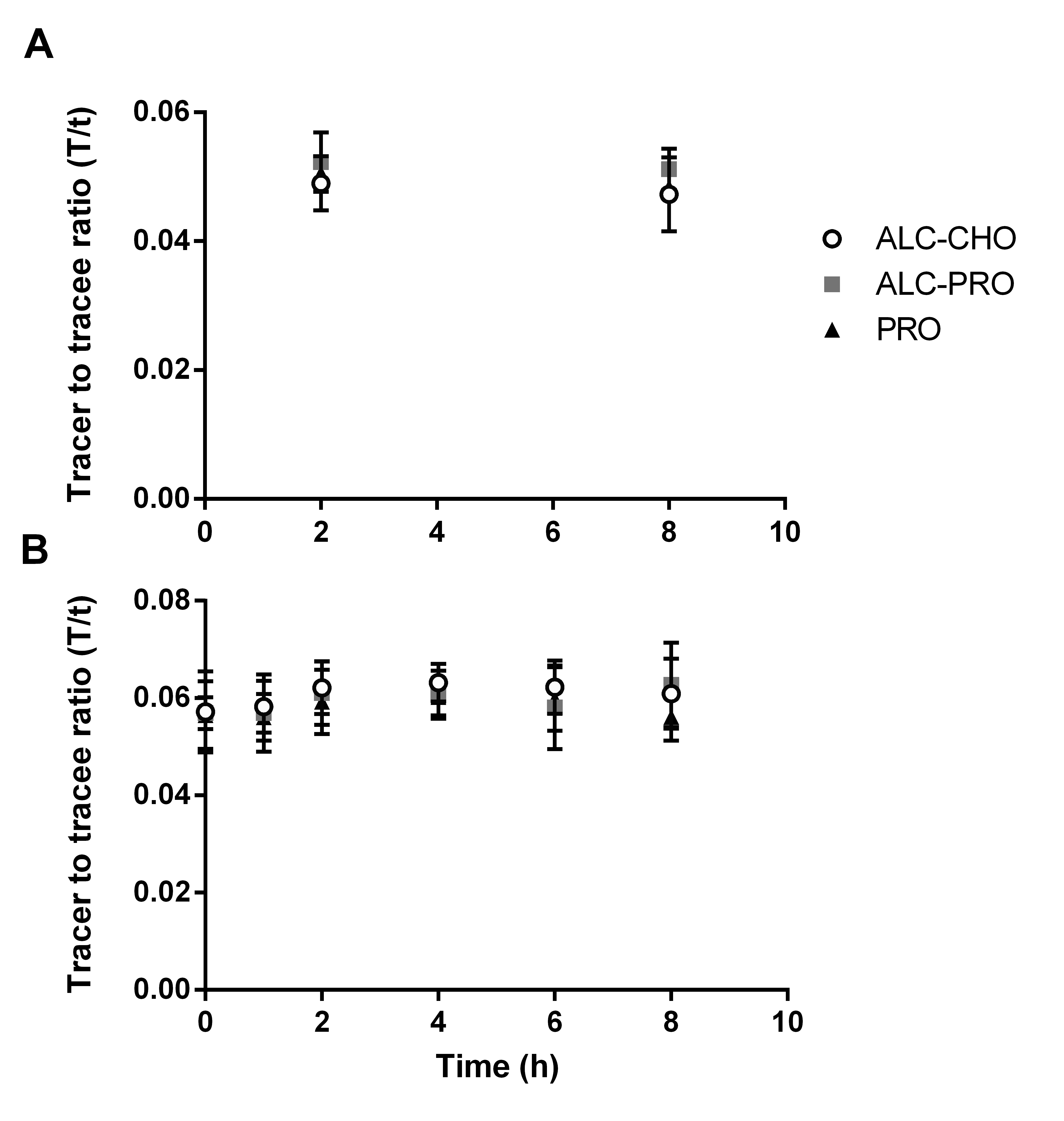

Supplement: Figure S1 — Tracer enrichment of the muscle intra-cellular protein pool (A) and blood plasma (B) following a single bout of concurrent training. (TIF) [file pone.0088384.s001.tif]
